# Vegetable Oil as a Carbon Resource and Growth Elicitor for the Liquid Fermentation of *Poria cocos*

**DOI:** 10.3390/jof11110815

**Published:** 2025-11-17

**Authors:** Biaobiao Luo, Rudan Wei, Linghui Meng, Nokwanda P. Makunga, Xuebo Hu

**Affiliations:** 1 College of Pharmacy, Henan Medical College, Xinxiang 453003, China; 211056@xxmu.edu.cn (B.L.); 222004@xxmu.edu.cn (L.M.); 2 Institute of Nanfan and Seed Industry, Guangdong Academy of Sciences, Guangzhou 510316, China; 18208941960@163.com; 3 Department of Botany and Zoology, Stellenbosch University, Private Bag X1, Matieland 7600, South Africa; makunga@sun.ac.za; 4 Laboratory of Natural Medicine and Molecular Engineering, College of Plant Science and Technology, Huazhong Agricultural University, Wuhan 430070, China

**Keywords:** *Poria cocos*, mycelia, fermentation, biomass, triterpenoid, vegetable oil, olive oil

## Abstract

Vegetable oil is a carbon-rich resource applied in liquid fermentation for compounds of interest. In this study, olive oil demonstrated the best effect on improving the liquid fermentation of a medicinal fungus *Poria cocos* (Schw.) Wolf compared to rapeseed, coix seed, palm, peanut, and soybean oils. When 2% (*v*/*v*) olive oil was initially added to the medium, biomass reached a maximum value of 11.7 g L^−1^, presenting a 3.1-fold enhancement compared to the blank control. Due to the stronger basal metabolism, the total triterpenoid yields also exhibited a significant improvement of ~3.4-fold, reaching 0.68 g L^−1^. Spectrophotometry, along with fluorescence and chemiluminescence probe assays, demonstrated that olive oil affected the fungus membrane fluidity and level of reactive oxygen species and nitrogen oxide in mycelium cells. Transcriptome analysis confirmed that olive oil was used as a carbon resource and elicitor that affected mycelia growth, which simultaneously produced some slight effects on metabolic processes, including fatty acid degradation, TCA cycle, and glycolysis/gluconeogenesis. Our study represents an attractive strategy for the industrial fermentation of filamentous fungi.

## 1. Introduction

*Poria cocos* (Schw.) Wolf is a filamentous fungus that grows as a saprophyte on a diverse range of *Pinus* spp. [1]. Its sclerotium, called fu-ling or hoelen, is used in traditional Chinese and Japanese medicine for its diuretic, sedative, and tonic effects [2]. In addition, with outstanding nutritional and health benefits, *P. cocos* has gained wide popularity as a nutraceutical and functional food in China [3]. In *P. cocos*, triterpenoids and polysaccharides are thought to be the major biochemical components, responsible for the pharmacological effects of *P. cocos* [4]. In particular, *P. cocos* triterpenoids have been shown to present inhibitory effects on inflammation, virus activation, cancer cells, and DNA polymerases [5]. As is well known, the triterpenoids of *P. cocos* are derived from lanostane or secolanostane skeletons [6], and their synthesis occurs via the mevalonate pathway [7].

In recent decades, the fermentation of filamentous fungus has been developed because of the industrial potential to efficiently obtain biological materials in a controlled environment, allowing for large scale access to pharmacologically important metabolites [8]. Liquid fermentation of *P. cocos* thus offers an economically viable solution as it can substantially reduce the total production period and significantly increase the productivity of mycelial biomass [9]. Owing to these advantages, it is therefore continually developed as an alternative strategy to produce compounds of interest. To improve fermentation, many studies have been conducted to examine the influence of medium composition on fermentation performance, including biomass accumulation and the content of bioactive compounds [10]. In particular, glucose is a common carbon and energy source, and it directly affects the growth of many secondary metabolite-producing microorganisms [11]. However, the rapid catabolism of glucose causes a decrease in the accumulation of biomass, thus limiting the biosynthesis of secondary metabolites [12]. In the liquid fermentation of multiple filamentous fungi, the positive effects of vegetable oils and fatty acids on biopolymer production and mycelial growth have been of interest, and several authors have indicated the usefulness of exploiting lipid-based agents for fungal culture [12,13,14,15]. Also, the addition of cottonseed oil, peanut oil, and essential oil has been employed in the liquid fermentation of filamentous fungi like *Cordyceps militaris* and *Antrodia cinnamomea* [15,16]. This approach utilizes vegetable oils as an effective carbon source. In previous studies, the addition of vegetable oil has been demonstrated to be favorable to mycelial growth in several filamentous fungi with increased production of bioactive metabolites [14,17]. Moreover, catabolite repression can be avoided by adding vegetable oils as carbon sources due to their low solubility in culture medium [18]. An additional benefit of vegetable oil is that it controls foaming [19]. The combination of plant oils and fatty acids with other carbon sources triggers exopolysaccharide accumulation and cell differentiation in *Grifola frondosa* [20], *Inonotus obliquus* [21], *Cordyceps militaris* [15], and *Ganoderma lucidum* [13]. Fatty acids play important roles in energy requirements and signaling cascades in the cell [22]. In addition, cyclic and acyclic products generated during fatty acid metabolism can also function as important chemical signals [23].

Previous studies have tried to explain how the application of exogenous oil affects the liquid fermentation of filamentous fungi from a biochemical level and genetic level, such as membrane fluidity, key gene and enzyme expression, important signal molecules, and metabolite synthesis [15]. Next-generation sequencing technology has been widely applied in the analysis of transcriptomes, biological characteristics, and functional genes in various fungi, such as *P. cocos* [24], *G. lucidum* [25], and *Codonopsis pilosula* [26]. Transcriptomics provides a powerful tool to explore the complex mechanism(s) relevant to biochemical behaviors at the system level [27], providing insights into the regulatory network that specifically controls specialized metabolism. To our knowledge, few studies have used RNA-seq to analyze the deep network mechanisms of gene expression after the addition of vegetable oils in the liquid fermentation of filamentous fungi.

Although there are a few studies on the effect of vegetables oils in the biosynthesis of non-polysaccharide secondary metabolites, information is still largely lacking in *P. cocos*. In this particular study, the strategy of adding vegetable oil to the medium was applied to improve the liquid fermentation of *P. cocos* with respect to biomass and triterpenoid content. The potential mechanism of metabolic regulation on central metabolic pathways, mycelium growth, and triterpenoid biosynthesis was also investigated by differential transcriptome analysis using a next-generation RNA-seq approach.

## 2. Materials and Methods

### 2.1. Chemicals

Six common vegetable oils were collected. Olive oil and rapeseed oil were purchased from Yihai Kerry Food Marketing Company (Beijing, China). Coix seed oil was purchased from Greeno Co., Ltd. (Beijing, China). Palm oil was purchased from Angel Yeast Company (Yichang, China). Peanut oil was purchased from Luhua Group Co., Ltd. (Laiyang, China). Soybean oil was purchased from Jinxin Agricultural Technology Development Co., Ltd. (Leiyang, China). Dihydrorhodamine 123 (DHR 123) was obtained from Shanghai Yuanye Bio-Technology Co., Ltd. (Shanghai, China). The other reagents were bought from Sangon Biotech Co., Ltd. (Shanghai, China).

### 2.2. Strain, Media, and Fermentation

*P. cocos* strain PCJzh001 was kept in our laboratory. The stock culture was maintained on potato dextrose agar (PDA), containing 200 g L^−1^ of potato extract, 20 g L^−1^ of glucose, and 13 g L^−1^ of agar. The inoculated plate was incubated at 28 °C for 7 days and then stored at 4 °C for pre-culture. Scale-up cultivation was performed with potato dextrose broth (PDB). The fermentation medium was composed of 35 g L^−1^ of glucose, 5 g L^−1^ of peptone, 2.5 g L^−1^ of yeast extract, 0.5 g L^−1^ of MgSO_4_·7H_2_O, 0.883 g L^−1^ of KH_2_PO_4_, and 0.05 g L^−1^ of vitamin B1.

Forty agar disks (0.5 cm) covered with *P. cocos* mycelia were excised from a PDA plate using the wide end of a 1 mL tip before these were transferred into a 250 mL flask containing 100 mL of PDB medium. The pre-cultures were shaken at 28 °C and 150 rpm for 5 days. The first seed cultures were grown in 250 mL flasks containing 40 mL of fermentation medium inoculated with 10 mL of pre-cultures at 150 rpm and 28 °C for 5 days. The second seed cultures were then grown in 250 mL flasks containing 45 mL of fermentation medium inoculated with 5 mL of first seed cultures at 150 rpm and 28 °C for 5 days. The fermentation for screening vegetable oils was performed in 250 mL flasks containing 100 mL of fermentation medium inoculated with 1 mL of second seed cultures at 150 rpm and 28 °C for 6 days. Vegetable oils of 1% (*v*/*v*) were added to the medium on day 0. The optimal fermentation was performed in 250 mL flasks containing 92 mL of fermentation medium inoculated with 8 mL of second seed culture at 150 rpm and 28 °C for 14 days. A final concentration of 2% olive oil (*v*/*v*) was added to the fermentation medium on day 0.

### 2.3. Measurement of Biomass

After the liquid fermentation of *P. cocos* was finished, the fermentation broth was centrifuged to obtain precipitates of mycelia, which were subsequently transferred to filter paper and washed using distilled water. After filtration, the pellets of mycelia were then dried to a constant weight in an oven at 50 °C. The biomass was determined by dry weight of mycelia pellets on an electronic balance.

### 2.4. Measurement of Triterpenoid

Following a previous protocol established in our laboratory [28], triterpenoids were extracted from *P. cocos* mycelia. Briefly, 50 mg of mycelia powder was sonicated in 3 mL 50% (*v*/*v*) twice (each time for 2 h). The supernatants were dried at 50 °C using a rotary evaporator. The residue was then resuspended in 3 mL of water and extracted with chloroform twice. The chloroform layer was dried at 45 °C using a rotary evaporator. Once evaporated, the extract residues were dissolved in 3 mL of methanol.

The concentration of triterpenoid in the extraction examples was measured using Wei’s method with a slight modification [29]. Briefly, after a 100 μL sample solution was heated to evaporation in a water bath, 300 μL of fresh 5% (*v*/*v*) vanillin–acetic solution and 1 mL of sulfuric acid were added and then incubated at 70 °C for 20 min. The cooled solution was diluted to 5 mL with acetic acid. The control experiment included all steps except for the addition of the sample solution. The optical absorption of the solution at 550 nm was measured against the control using a spectrophotometer. The triterpenoid content in the sample power, expressed as a percentage, was determined using the standard oleanolic acid calibration curve in a range of 0.1–0.6 mg mL^−1^ (Y = 0.2781X + 0.0351, r^2^ = 0.9981; X, absorbance value; Y, concentration). Triterpenoid yield was calculated based on values of biomass and triterpenoid and expressed as milligram oleanolic acid equivalent per liter of fermentation broth.

### 2.5. Measurement of Nitride Oxide (NO)

The total NO in *P. cocos* mycelia was measured calorimetrically using a commercial Total Nitric Oxide Assay Kit (Bi Yun Tian, Nanjing, China). Mycelia of *P. cocos* (0.1 g) were completely crushed by grinding over ice and then resuspended in 1 mL of double-distilled H_2_O (ddH_2_O), followed by the removal of cell lysate by centrifugation. A total of 100 μL of supernatant was mixed with 100 μL of Griess reagents. An equal volume of ddH_2_O was used as the blank control. The optical absorption of the mixtures at 550 nm was measured against the blank control using a spectrophotometer. The NO content was determined according to the standard NaNO_2_ calibration curve in a range of 10~100 μM (Y = 0.0109X + 0.0273, r^2^ = 0.9983; X, NaNO_2_ concentration; Y, absorption value) and expressed as millimoles NaNO_2_ equivalent per gram of *P. cocos* mycelia.

### 2.6. Measurement of Reactive Oxygen Species (ROS)

The ROS content was measured as follows: 0.3 g of *P. cocos* mycelia was slightly ground over ice and then resuspended in 6 mL of phosphate-buffered saline (PBS) solution, followed by removal of cell lysate by filtration with a 300 mesh × 83 μm nylon membrane (Merck, Darmstadt, Germany). For each measurement, a sample solution (500 μL) was mixed with 1 μL of DHR 123 (1 mg mL^−1^), followed by a 28 °C incubation for 15 min. Finally, the fluorescence intensity of ROS in the mixture was detected using a Guava EasyCyte 8 system (Millipore, Burlington, MA, USA) via flow cytometry (Millipore).

### 2.7. Measurement of Membrane Fluidity

The membrane fluidity of *P. cocos* mycelia was determined by fluorescence anisotropy following Liu’s protocol [30] with some modifications. The fresh mycelia on day 6 of fermentation were kept in a fixation solution (10.93% (*v*/*v*) mannitol, 0.25% (*v*/*v*) methanol) for 1 h. Subsequently, fixed mycelia were incubated at 30 °C in lysing solution (2% lysing enzymes, 10.93% (*v*/*v*) mannitol) for 1.5 h. After centrifugation, cells were washed twice with PBS (pH 7.4) containing 0.25% (*v*/*v*) methanol. Thereafter, incubation with probe solution (5 μM 6-diphenyl-1,3,5-hexatriene) was performed at 37 °C for 1 h. After removal of the free probe by centrifugation, cells were resuspended in PBS (pH 7.4) to a final optical density of 0.6 at 600 nm. Fluorescence anisotropy was measured at 37 °C using a spectrofluorometer with excitation at 360 nm and emission at 430 nm (5 and 5 nm slits, respectively). Anisotropy values (r) were calculated as [*I*_VV_*-I*_VH_(*I*_HV_/*I*_HH_)]/[*I*_VV_*+*2*I*_VH_(*I*_HV_/*I*_HH_)], where *I* is the corrected fluorescence intensity, and the subscripts V and H indicate the values obtained with vertical or horizontal orientations, respectively, of the excitation polarizer and emission analyzer (in that order).

### 2.8. RNA Extraction and Sequencing

Total RNA was extracted from *P. cocos* cells using TRIzol^®^ Reagent following the protocol designed by the manufacturer (Invitrogen, Carlsbad, CA, USA). The qualified RNA sample was identified for transcriptome sequencing. An RNA sample preparation kit (Illumina, San Diego, CA, USA) was used to construct an RNA library. Firstly, mRNA with poly-A tails was enriched from 5 μg of total RNA using oligo-dT magnetic beads and then fragmented randomly into snippets with 200 bp by fragmentation buffer. Subsequently, cDNA was synthesized from mRNA templates, using the SuperScript double-stranded cDNA synthesis kit (Invitrogen) with the addition of random hexamer primers (Illumina). Double-stranded cDNA was processed with end-repair and ‘A’ base addition. Eventually, the cDNA was amplified by PCR, and 200~300 bp bands were recovered using 2% agarose. The Illumina HiSeq2500 sequencing platform was used for high-throughput sequencing.

### 2.9. Statistical Analysis

All data obtained in this work were the means of triplicate experiments and expressed as mean ± SD. One-way ANOVA was used to analyze the data. Statistical significance was defined as *p* < 0.05.

## 3. Results

### 3.1. Effect of Various Vegetable Oils on the Liquid Fermentation of P. cocos

**Figure 1:**
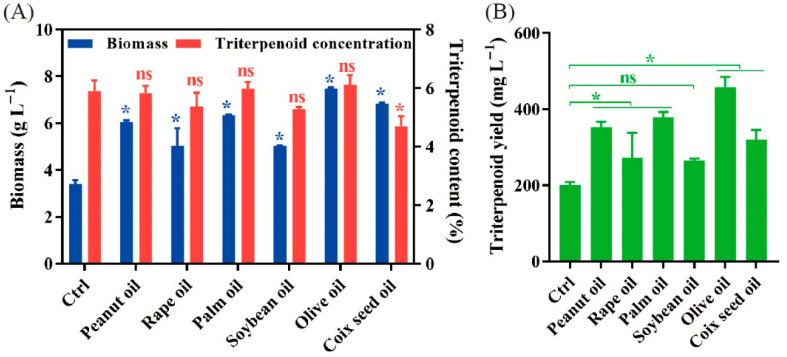
The effects of various vegetable oils on the biomass and triterpenoid content (**A**) and triterpenoid yield (**B**) in the fermentation of *P. cocos*. The fermentation without oil addition is used as the control. All data are from triplicates (means ± SD). Asterisks denote (* *p* < 0.01) statistically significant differences between the treatment group and the control; ns indicates no significant difference.

In this study, how vegetable oils affect mycelial growth and triterpenoid production in liquid fermentation of *P. cocos* was primarily analyzed as follows: Various vegetable oils of 1% (*v*/*v*) were added in fresh fermentation medium as the secondary carbon sources for mycelial cells in liquid fermentation of *P. cocos*, and the medium without oil was set as the control. Adding vegetable oils significantly led the biomass of mycelia to increase 1.5~2.2-fold (Figure 1A). Maximum biomass was achieved by olive oil followed by coix seed oil. Fungus biomass after the addition of palm oil and peanut oil was lower than that for coix oil. Rapeseed oil and soybean oil showed the lowest increases in mycelium biomass. On the other hand, adding vegetable oils not only had no positive impact on the triterpenoid content but adding coix seed oil actually decreased it (Figure 1A). These phenomena suggest that vegetables oils mainly contribute to the growth of *P. cocos* mycelia. Eventually, the addition of vegetable oils significantly increased the total triterpenoid yield 1.3~2.3-fold, and olive oil was demonstrated to have the greatest effect (Figure 1B).

### 3.2. Optimization of Olive Oil in the Fermentation of P. cocos

**Figure 2:**
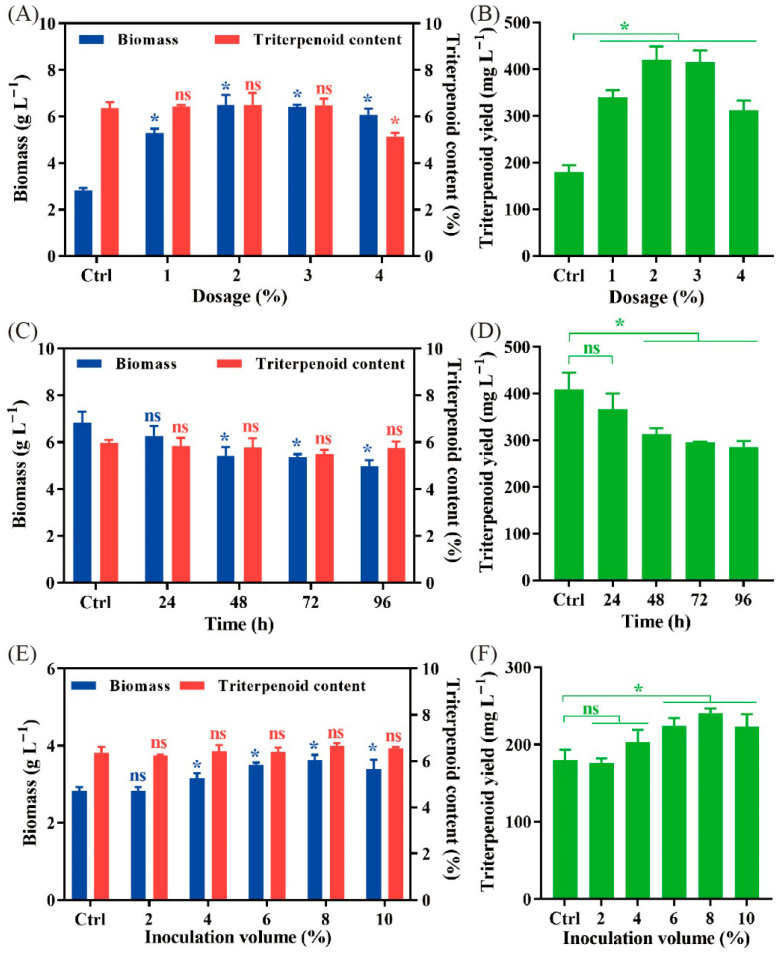
The optimization of liquid fermentation of *P. cocos*. The effect of oil dosage, addition time, and inoculation volume on biomass and triterpenoid content (**A**,**C**,**E**) and triterpenoid yield (**B**,**D**,**F**) was quantitatively analyzed. Liquid fermentation without oil (**A**,**B**), with 2% (*v*/*v*) olive oil (**C**,**D**), or 1% (*v*/*v*) inoculation (**E**,**F**) as control. All data were from triplicates (means ± SD). Asterisks denote (* *p* < 0.05) statistically significant differences between treatment groups and the control; ns indicates no significant difference.

To further improve the performance of olive oil in the liquid fermentation of *P. cocos*, its dosage and addition time were deeply investigated. The dosage of olive oil was titrated in a range of 1~4% (*v*/*v*), and addition without oil was used as the control. Figure 2A shows that all dosages promoted biomass accumulation. However, the magnitude of this promotion exhibited a dose-dependent decrease. A dosage of 2% enabled the biomass to reach its maximum value of 6.49 ± 0.44 g L^−1^. The dosage effect on the triterpenoid content was also determined. There was no difference in the dosages of 1~3%, and triterpenoid content was significantly decreased by a higher dosage of 4% (Figure 2A). Consequently, the maximum triterpenoid yield was observed at a dosage of 2% olive oil (Figure 2B), which was considered the best concentration in the optimal fermentation of *P. cocos*.

Subsequently, the best addition time of olive oil was determined. Olive oil with an optimal concentration of 2% (*v*/*v*) was added at a time point ranging between 0 and 96 h, and addition at 0 h was used as the control. It was proven that the biomass of *P. cocos* mycelia presented a decreasing trend along with the delay of addition time, and the addition at 8 h achieved the highest biomass, which was increased 2.5-fold compared to the control (Figure 2C). Meanwhile, the timing of olive oil addition did not affect the triterpenoid content in submerged mycelia (Figure 2C). Therefore, when the olive oil was added at 0 h, the maximum triterpenoid yield was obtained (Figure 2D). In conclusion, the best addition time of olive oil was at 0 h, which was applied in the subsequent experiments.

We then investigated the inoculation volume of the second seed culture, which was varied in the range of 1~10% (*v*/*v*), and the inoculation of 1% (*v*/*v*) was used as the control. When the fermentation was over, there was no difference in triterpenoid content after fermentation with different volumes of inoculation (Figure 2E). However, the biomass was significantly improved with an inoculation concentration higher than 4% (*v*/*v*) (Figure 2E). When 8% (*v*/*v*) second seed culture was inoculated into the medium, mycelium biomass and triterpenoid yield reached their peak values (Figure 2E,F). Consequently, the inoculation volume of 8% (*v*/*v*) was used in the next optimal liquid fermentation of *P. cocos*.

### 3.3. The Optimal Liquid Fermentation of P. cocos

**Figure 3:**
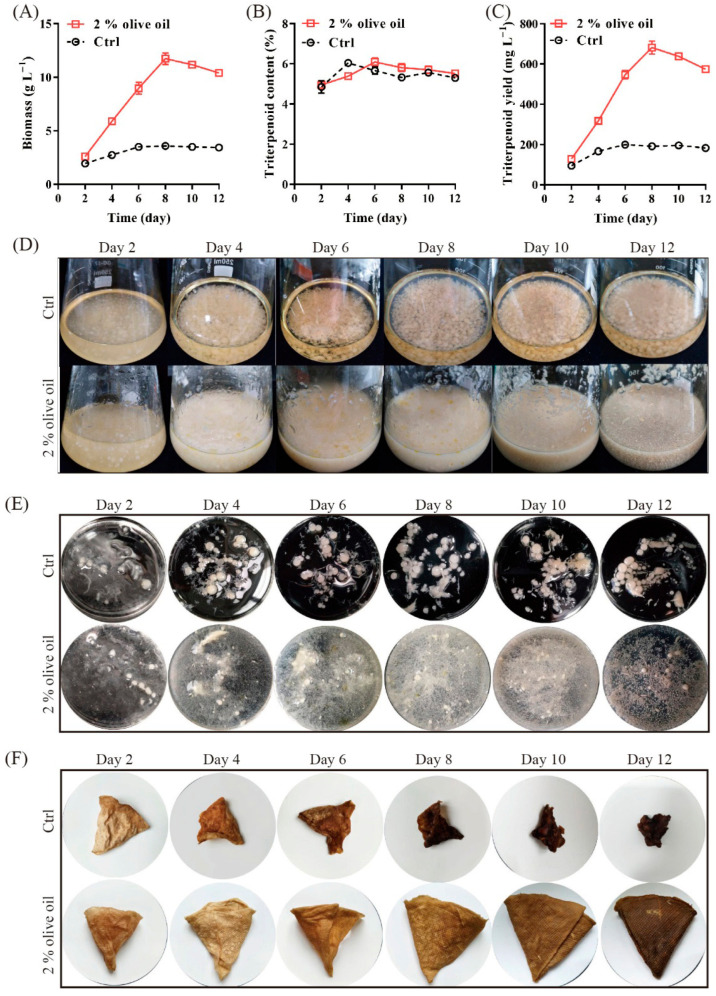
The progress of optimal liquid fermentation of *P. cocos*. The biomass (**A**), triterpenoid content (**B**), and triterpenoid yield (**C**) were quantitatively analyzed. Graphs of fermentation broths (**D**), mycelia aggregates (**E**), and dried mycelia pellets (**F**) are presented along with time. The liquid fermentation without oil is used as the control. All data are from triplicates (means ± SD).

Based on the results above, an optimal liquid fermentation of *P. cocos* was conducted for 12 days with an addition of 2% (*v*/*v*) olive oil and an initial inoculation of 8%, and fermentation in medium without oil was used as the control. Olive oil addition elicited a rapid increase from day 1 to day 8, followed by a gradual decrease (Figure 3A). On day 8, the biomass reached the highest level of 11.74 ± 0.54 g L^−1^, which was about 3.3-fold higher than that of the control (3.60 ± 0.12 g L^−1^) on day 6 (Figure 3A). On the other hand, the triterpenoid content did not exhibit an obvious difference compared to the control (Figure 3B). Eventually, the triterpenoid yield reached a maximum level of 682.5 ± 30.0 mg L^−1^, which was 3.4-fold higher than the highest point (199.3 ± 13.5 mg L^−1^) on day 6 in the control (Figure 3C).

Mycelia morphology is an indicator of the growth status of *P. cocos*. The fermentation broth with oil started to become cloudy on day 2, while the control remained clear during the whole fermentation process (Figure 3D). Under the microscope, it was shown that the mycelia were more evenly dispersed in the media than the control, while mycelia in the control tended to form into balls (Figure 3D,E). Changes in biomass were qualitatively assessed by observing the size of the dried mycelial pellets. Those pellets formed by the addition of olive oil appeared obviously bigger (Figure 3F).

**Figure 4:**
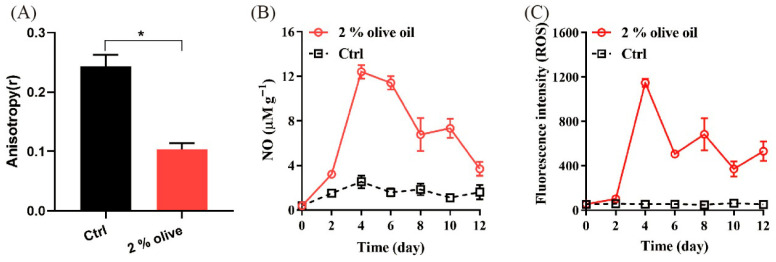
The membrane fluidity (**A**), NO level (**B**), and ROS level (**C**) of cells in the optimal fermentation of *P. cocos*. Liquid fermentation without oil is used as the control. The data indicate the means ± SD of triplicates. Asterisks denote (* *p* < 0.05) statistically significant differences between treatment group and the control.

We demonstrated that supplementing the medium with 2% olive oil significantly reduced the fluorescence anisotropy of mycelial cells (Figure 4A), indicating an increase in membrane fluidity compared to the oil-free control. Furthermore, oil addition enhanced the accumulation of key signaling molecules NO (Figure 4B) and ROS (Figure 4C) compared to the control. The levels of NO and ROS showed a similar temporal trend: both rose gradually from day 0 to day 2, increased more rapidly until day 4, and then declined gradually throughout day 12. Notably, both molecules remained at significantly higher concentrations in the olive oil treatment than in the control at the end of the 12 day period (Figure 4B,C).

### 3.4. Differential Transcriptome Analysis

In order to better understand the effect of olive oil addition on gene expression during fermentation by *P. cocos* mycelia, time-resolved transcriptome analysis was carried out. The *P. cocos* mycelia were collected in the optimal fermentation and the control on days 4 and 8 (with three biological replicates each), and the samples were then used for RNA extraction and transcriptome sequencing. In total, transcriptome analysis of 12 samples was completed and 58.77 Gb of clean data was collected. The clean data of each one was more than 4.20 Gb, and the percentage of Q30 bases was more than 94.86%. Moreover, the alignment rates ranged from 80.68% to 84.87%, showing a good transcriptome assembly quality.

**Figure 5:**
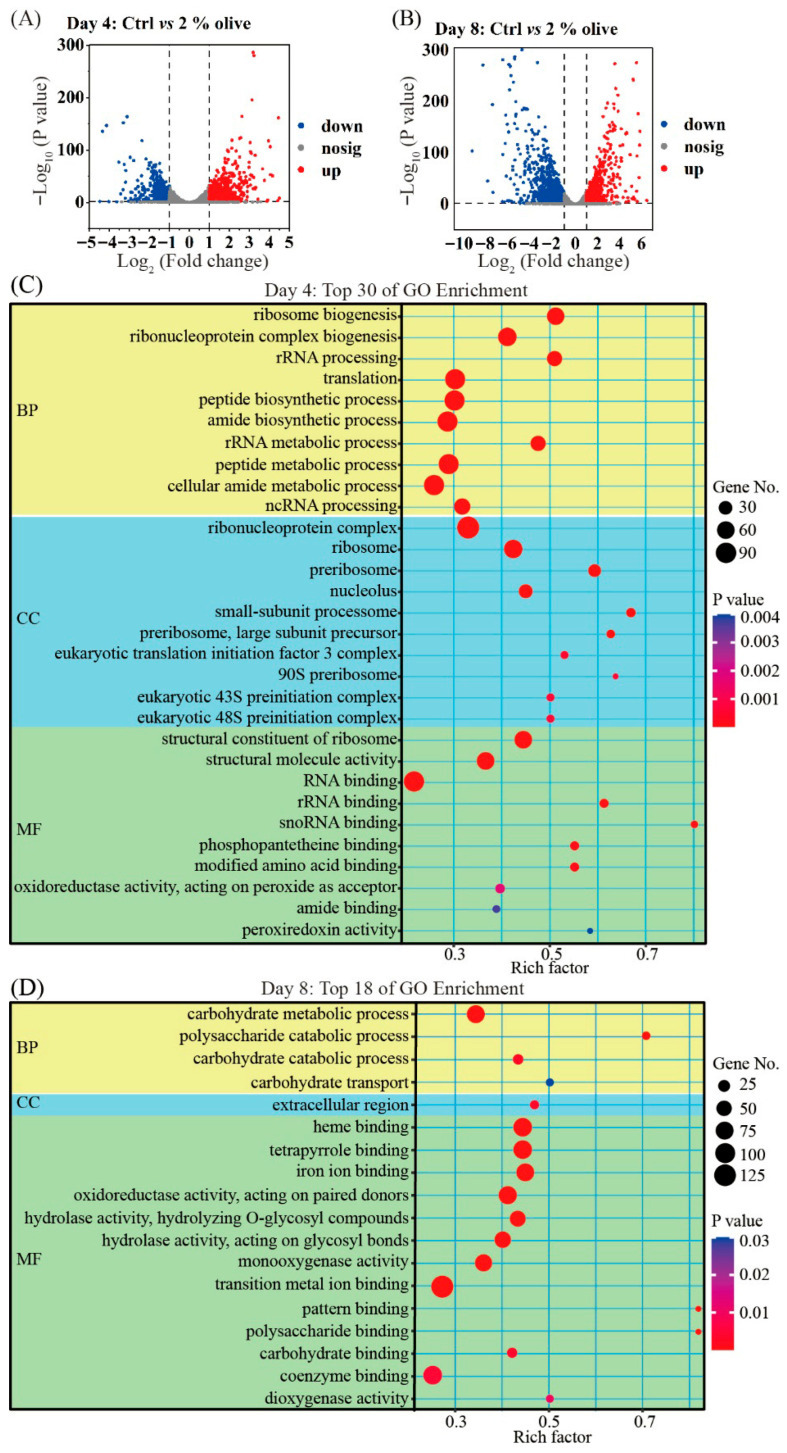
Volcano plots of DEGs on day 4 (**A**) and day 8 (**B**) between the optimal liquid fermentation and the control. Liquid fermentation without oil is used as the control. (**C**) Top 30 significant (*p* < 0.05) GO terms of DEGs on day 4 are indicated. (**D**) All significantly enriched (*p* < 0.05) GO terms of DEGs on day 8 are presented.

Figure 5A,B show differences in gene expression in mycelia between optimal liquid fermentation and the control on days 4 and 8. We identified a total of 1430 (including 736 up- and 694 downregulated) differentially expressed genes (DEGs) on day 4 (Figure 5A) and a total of 1972 (including 788 up- and 1184 downregulated) DEGs on day 8 (Figure 5B). We wondered if olive oil would have a greater effect on biological processes/metabolism during the earlier or later stages of fermentation. Therefore, we performed GO enrichment analysis for these DEGs (Figure 5C,D). Generally, GO analysis for DEGs on day 4 identified enrichment for 35 biological process (BP), 13 cell component (CC), and 13 molecular function (MF) terms, and the top 30 enriched GO terms are shown in Figure 5C. On day 8, GO analysis indicated that many differentially expressed transcripts had roles, relating to secondary metabolism (Figure 5D), indicating that olive oil played a key role in metabolism during the later period.

**Figure 6:**
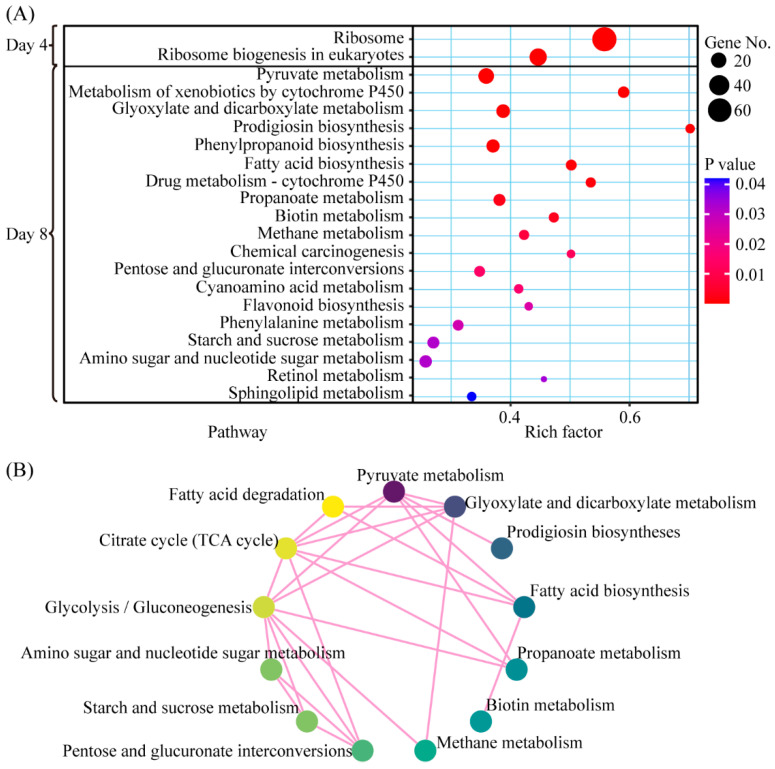
(**A**) All significant (*p* < 0.05) KEGG pathways on day 4 and day 8 between the optimal liquid fermentation and the control are indicated. Liquid fermentation without oil is used as the control. (**B**) The network of KEGG pathways relevant to fatty acid degradation, TCA cycle, and glycolysis/gluconeogenesis is shown.

To further identify the biological pathways activated by olive oil, we mapped DEGs to the reference canonical pathways in the KEGG database. On day 4 of fermentation, 63 and 27 DGEs were mapped to the KEGG terms ribosome (ko03010) and ribosome biogenesis in the eukaryotes pathway (ko03008), respectively, with significant enrichment in both cases (Figure 6A). These results imply that olive oil may promote enhanced peptide synthesis in fermenting *P. cocos*. On day 8, 183 DGEs were significantly enriched for 19 different pathways, including pyruvate metabolism (ko00620), metabolism of xenobiotics by cytochrome P450 (ko00980), glyoxylate and dicarboxylate metabolism (ko00630), prodigiosin biosynthesis (ko00333), and phenylpropanoid biosynthesis (ko00940) (Figure 6A). These results were able to provide insights into the influence of olive oil on secondary metabolism, and this was a time-dependent effect. A map of the KEGG network indicated that most of the identified pathways were crucial to glycolysis/gluconeogenesis (ko00010), citrate cycle (ko00020), and fatty acid degradation (ko00071) (Figure 6B).

## 4. Discussion

In this research, the observed enhancement of biomass and triterpenoid yield in *P. cocos* following olive oil supplementation is consistent with the findings in other basidiomycetes. For instance, studies on *Ganoderma lucidum* [13], *Cordyceps militaris* [15], and *Antrodia camphorate* [16] have similarly reported that lipid-rich substrates can increase biomass or triterpenoid yields. For practical scale-up, the cost of olive oil versus glucose must be considered. Although olive oil is more expensive per unit, our data show that it supports higher mycelial growth and triterpenoid yield per substrate, which may balance out its initial cost. While a detailed techno-economic analysis is needed for industrial translation, our findings support olive oil as an effective strategy for enhancing triterpenoid production in *P. cocos*.

Vegetable oils are mainly composed of triglycerides (95–96%). The molecular structures of triglycerides in vegetable oils are very important for filamentous fungal fermentation when oils are used for the exogenous addition [13]. The length of carbon chain and the extent of unsaturation of fatty acids in vegetable oils determine the extent of stimulation or suppression in metabolite production [15]. In addition, fatty acids are known to have a regulatory effect by participating in central and specialized metabolisms in eukaryotes [31]. So, the enhancing effect of olive oil on the growth of *P. cocos* mycelia (Figure 1A) seems to be linked to its own characteristics related to fatty acid composition and unsaturation. The composition of olive oil is complex, with multiple fatty acids, including palmitic (C16:0), palmitoleic (C16:1), stearic (C18:0), oleic (C18:1), linoleic (C18:2), and linolenic (C18:3) types [32], and they exhibit the highest level of unsaturation. This may be the key to the better effect of olive oil compared to the other vegetable oils.

Fatty acids are essential structural components of cells [22], and membrane fluidity is known to be influenced by membrane fatty acid composition [33]. The mechanism by which olive oil promotes growth may involve the incorporation of its unsaturated fatty acids [32] into the cell membrane. This incorporation, previously suggested to enhance membrane fluidity and nutrient uptake [34,35], is directly supported by our observed decrease in fluorescence anisotropy (Figure 4A), which indicates a corresponding increase in membrane fluidity. The increased membrane fluidity possibly promotes membrane permeability and the transport and assimilation of carbon and nitrogen resources, salts, and other growth-promoting factors. In *P. cocos* cells, the sudden increase in exogenous chemicals likely induces a stress response. This is evidenced by the elevated levels of reactive oxygen species (ROS) and nitric oxide (NO) (Figure 4B,C), which may function as signaling molecules to initiate downstream cellular defense programs. Therefore, lipids and fatty acids can act as both intracellular and extracellular signals. Moreover, mitochondrial fatty acid oxidation is the source of increased net ROS production [36]. So, the increased ROS level (Figure 4B) partially supports the hypothesis that olive oil as a carbon source participates in the energy supply for the growth of *P. cocos* mycelia.

A previous study showed that in addition to sugars, exogenous lipids are a major source of organic carbon delivered to the fungus, and this is necessary for the production of fungal lipids [37]. Fatty acid degradation in most organisms occurs primarily via the β-oxidation cycles, and most fungi harbor the β-oxidation cycle only in the peroxisomes [38]. Uptake of fatty acids allows for the organism to survive nutrient deprivation conditions by upregulating fatty acid β-oxidation, which can provide more energy and coenzymes for cell growth [39,40]. The RNAseq results indicated that six genes in the fatty acid β-oxidation pathway were upregulated on day 4 and day 8 during the oil fermentation (Table 1), demonstrating that olive oil oxidation is likely accelerated to promote mycelial growth. In this particular study, GO and KEGG enrichment analyses showed that a large cohort of DGEs induced by oil are involved in the biosynthesis pathways and biological processes that manufacture proteins and nucleic acids. Differential expressions elicited through modifying the growth conditions of *P. cocos* allow for increased production of primary building blocks and provide a rich chemical foundation for mycelial growth.

RNA-seq confirmed that genes associated with metabolism of ROS and NO are upregulated during oil fermentation (Table 2). The mechanisms that control stress moderation in biological systems are complex. In many different organisms, including fungi, ROS and NO act via secondary messengers (Ca^2+^, cAMP, IP3, arachidonic acid) with their specific targets, including ion channels, transporters, kinases, phosphatases, and transcription factors [41]. Thus, they are implicated in the biochemical and genetic adjustments of plants to stress. Both ROS and NO signaling generally influence specialized metabolism and those pathways that are involved in triterpenoid synthesis, which appear to be activated.

## 5. Conclusions

In this study, olive oil was employed to improve growth productivity in the fermentation of *P. cocos* mycelia; its application increased the biomass of submerged mycelia 3.3-fold, reaching 11.74 ± 0.54 g L^−1^. As a result, the operation of adding olive oil to the medium indirectly influenced triterpenoid production during liquid fermentation of *P. cocos*, and its yield was increased 3.4-fold, corresponding to 682.5 ± 30.0 mg L^−1^. It is thus likely that olive oil exhibits its beneficial effects through increasing membrane fluidity and permeability; this may be connected to increasing levels of ROS and NO, which are important signaling molecules closely related to the central metabolism of carbon, RNA transcript, and protein synthesis. As a safe elicitor and carbon resource and a relatively simple technology, the addition of vegetable oils exhibits a promising prospect for large-scale industrial production of biomass and terpenoids using *P. cocos* mycelia.

## Figures and Tables

**Table 1:** Expression of DGEs relevant to fatty acid oxidation.

Gene	Expression Abundance ^1^				Annotation
	Oil 4	Ctrl 4	Oil 8	Ctrl 8	
WC07318	3.50	3.14	3.63	3.07	Acetyl-CoA synthetase-like protein
WC09938	3.39	2.44	3.11	2.41	Acyl-CoA N-acyltransferase
WC08049	2.65	2.45	2.60	2.53	Putative acyl-CoA synthetase
WC08376	3.69	3.51	3.82	3.26	Acyl-CoA N-acyltransferase
WC06746	3.43	3.21	3.31	3.28	Acyl-CoA N-acyltransferase
WC00558	1.70	1.33	1.46	1.34	Acyl-CoA dehydrogenase domain-like protein
WC05710	3.45	3.57	3.62	3.37	Acyl-CoA dehydrogenase domain-like protein
WC02747	3.16	3.09	3.31	3.05	Acetyl-CoA synthetase-like protein

^1^ The data of expression abundance are the value of Log10 (TPM + 1). TPM indicates transcript per million.

**Table 2:** Expression of DGEs relevant to generation of ROS and RNS.

Gene	Expression Abundance ^1^				Annotation
	Oil 4	Ctrl 4	Oil 8	Ctrl 8	
WC11803	2.87	2.34	2.63	1.97	Mitochondrial external NADH dehydrogenase
WC10033	3.89	3.66	3.68	3.72	Succinate dehydrogenase iron–sulfur subunit
WC04516	4.07	3.79	3.95	3.80	Mitochondrial cytochrome c oxidase subunit
WC00331	3.95	2.70	3.78	2.01	Alternative oxidase
WC01542	4.03	3.85	4.00	3.89	NADPH oxidase
WC04138	2.88	2.85	2.82	2.84	Nitric oxide synthase-interacting protein

^1^ The data present the value of Log10 (TPM + 1). TPM indicates transcript per million.

## Data Availability

The original data presented in the study are openly available in NCBI at https://submit.ncbi.nlm.nih.gov/subs/sra/SUB15771214 (accessed on 12 November 2025).
